# Where You Look Matters for Body Perception: Preferred Gaze Location Contributes to the Body Inversion Effect

**DOI:** 10.1371/journal.pone.0169148

**Published:** 2017-01-13

**Authors:** Joseph M. Arizpe, Danielle L. McKean, Jack W. Tsao, Annie W.-Y. Chan

**Affiliations:** 1 Department of Neurology, University of Tennessee Health Science Center, Memphis, Tennessee, United States of America; 2 Le Bonheur Children's Hospital, Memphis, Tennessee, United States of America; 3 Department of Psychiatry, Harvard Medical School, Boston, Massachusetts, United States of America; 4 Boston Attention and Learning Laboratory, Boston Division Veterans Affairs Healthcare System, Jamaica Plain, Massachusetts, United States of America; 5 Children's Foundation Research Institute, Le Bonheur Children's Hospital, Memphis, Tennessee, United States of America; 6 Department of Anatomy & Neurobiology, University of Tennessee Health Science Center, Memphis, Tennessee, United States of America; 7 Memphis Veterans Affairs Medical Center, Memphis, Tennessee, United States of America; University of Muenster, GERMANY

## Abstract

The Body Inversion Effect (BIE; reduced visual discrimination performance for inverted compared to upright bodies) suggests that bodies are visually processed configurally; however, the specific importance of head posture information in the BIE has been indicated in reports of BIE reduction for whole bodies with fixed head position and for headless bodies. Through measurement of gaze patterns and investigation of the causal relation of fixation location to visual body discrimination performance, the present study reveals joint contributions of feature and configuration processing to visual body discrimination. Participants predominantly gazed at the (body-centric) upper body for upright bodies and the lower body for inverted bodies in the context of an experimental paradigm directly comparable to that of prior studies of the BIE. Subsequent manipulation of fixation location indicates that these preferential gaze locations causally contributed to the BIE for whole bodies largely due to the informative nature of gazing at or near the head. Also, a BIE was detected for both whole and headless bodies even when fixation location on the body was held constant, indicating a role of configural processing in body discrimination, though inclusion of the head posture information was still highly discriminative in the context of such processing. Interestingly, the impact of configuration (upright and inverted) to the BIE appears greater than that of differential preferred gaze locations.

## Introduction

Prior studies have indicated specialized visual processing of human bodies that involves neural substrates and perceptual mechanisms that are distinct from those involved in the processing of other object categories. For example, the Extrastriate Body Area (EBA) and Fusiform Body Area (FBA) are functionally-defined human brain regions that exhibit selective visual responses to human bodies or body parts relative to faces and other object categories [1–5]. Further, specialized processing mechanisms, much like those thought to be involved in face perception [6–10], are evident from behavioral studies reporting that visual discrimination performance for whole (i.e., head included, always without a face) inverted bodies is reduced compared to whole upright bodies, but that this Body Inversion Effect (BIE) is larger in magnitude than any inversion effects for other object categories, with the exception of faces [11–17]. Studies investigating the BIE as a means of uncovering the mechanisms of visual body perception have begun to elucidate the specific natures of these perceptual mechanisms.

For example, reports that the BIE is absent for isolated body parts, impossible body part configurations [12], and for unnatural body postures [11,18] suggest that some sort of configural (i.e., relating to feature arrangement information “more than the sum of its parts”) processing mechanisms analogous to those for processing faces [6–10] are implicated in the visual processing of whole bodies. Evidence indicates that any configural processing of whole bodies cannot be regarded as strictly holistic (i.e. not processed principally as a unitized, non-decomposable whole), but rather that head posture information is particularly important in the visual processing necessary for body posture discrimination. Specifically, a robust BIE has been reported for whole bodies and for bodies without either arms or legs, though a reduction or elimination of the BIE has been reported for whole bodies with fixed head position and for headless bodies [14,16]. The (faceless) head must be viewed in at least a partial body context in order to induce the BIE, while isolated faceless heads have not been reported to show an inversion effect [19].

Given the importance of the head posture information in the BIE and the similarity of the BIE to the face inversion effect, it has been hypothesized that face/head-selective neural substrates and their corresponding face/head-selective perceptual mechanisms drive the BIE. Evidence consistent with this hypothesis has come from a functional neuroimaging study [13] reporting that the face-selective fusiform face area (FFA), but not the body-selective EBA and FBA regions, showed an adaptation effect to upright but not inverted (faceless) bodies in the case of whole bodies but no adaptation in the case of headless bodies of either orientation. Thus, the adaptation pattern in a face selective area, and not in body-selective areas, was consistent with the BIE in the behavioral results for whole but not headless bodies. However, evidence against this hypothesis comes from a subsequent study on acquired prosopagnosic patients that reports that the face inversion effect, but not the BIE, was reduced in the patients compared to healthy controls [15]. Impairment in face identification mechanisms therefore did not seem to impact body processing. Further, a significant, though smaller, BIE for headless bodies was also reported for controls in the study, suggesting that a small inversion effect existed even in the absence of the head information. Additionally, another study [20] reported a BIE for piecemeal bodies without heads and trunks, but with the first-order spatial relations among limbs preserved. Given the incomplete understanding of the processing mechanisms involved in the BIE, and thus in body perception more generally, we sought to further elucidate these mechanisms.

The present study reveals the contributions of body feature and configuration processing to human visual body discrimination through two complementary measures not previously considered together in the context of the BIE. Specifically, we first investigate gaze patterns during a body posture discrimination task involving upright and inverted whole bodies (albeit with faceless heads) and headless bodies. Though one prior study measured eye-movements during body discrimination [18], the functional significance of those eye-movements was not tested. Further, fixation location was not controlled in prior studies of the BIE. Importantly, we therefore subsequently investigate the degree to which the observed fixation locations causally relates to discrimination performance by manipulating fixation location in a forced-fixation paradigm. Forced-fixation paradigms have been used successfully in some prior studies of face perception to study the functional significance of specific preferred fixation locations [21–26].

Our study specifically had three principal aims, namely (i) to test the hypothesis that differential locations of gaze to upright versus inverted bodies are causally implicated in the BIE under experimental conditions comparable to those of prior studies, (ii) to determine how, if at all, gaze patterns may relate to the reduction in the BIE for headless bodies compared to whole bodies as has been reported in prior studies, and (iii) determine if between upright and inverted bodies there are configural processing differences that are independent of gaze patterns. We report differential gaze patterns to upright versus inverted bodies, such that our participants predominantly gazed at the (body-centric) upper body for upright bodies, and at the lower body for inverted bodies. Subsequent controlled manipulation of fixation location allowed us to infer the functional significance of this specific pattern of differential gaze locations. Specifically, we found that it contributed to the BIE for whole bodies because of the informational value of the head posture for the body posture discrimination task. We also report that a BIE could be detected for both whole and headless bodies even when fixation location on the body was held constant, though inclusion of the head posture information was still highly discriminative in the context of such configural processing. Therefore, preferred gaze patterns causally influence the BIE, though the typical versus atypical configurations as such (i.e. upright and inverted) also drive the BIE. Interestingly, the influence of the differential configuration to the BIE appears greater than that of the differential preferred gaze locations.

## Materials and Methods

### Ethics statement

All participants gave written informed consent and were compensated for their participation. The study was approved by the Institutional Review Board of the University of Tennessee Health Science Center, Memphis, TN, USA.

### Participants

16 participants (4 male; 15 right-handed) aged 22–60 years (mean 34.7, standard deviation 12.5 years) are included in analyses. All had normal or corrected to normal vision. Participants attended all experimental sessions, allowing for within—subject analyses. We originally recruited 34 participants, but four were excluded because of poor eye-tracking calibration and six others were excluded because of poor fixation maintenance (< 50% of trials in at least one condition) in the relevant forced-fixation experiment sessions. The remaining eight volunteers presented scheduling difficulties, and so were excluded from analysis because they did not complete all four sessions.

### Eye-tracking

We used an EyeLink II headmounted eye-tracker (SR Research, Mississauga, ON, Canada), and sampled pupil centroid at 250 Hz during the trials of the experiments. Saccade sensitivity was set to “Normal” (i.e., 30°/sec velocity threshold and 8000°/sec^2^ acceleration threshold), link/analogue filter was set to “standard”, tracking mode was set to “pupil”, and file sample filter was set to “extra”. Participants’ eyes were 57 cm from the stimulus display screen. The default nine-point standard EyeLink^®^ calibration was performed for each participant at the start of each experimental session, and a validation sequence was also performed before each of the six experimental blocks (24 trials per block for Experiment 1 and 36 trials per block for Experiment 2). Both eyes were calibrated and validated, but only the eye with the lowest average error was recorded for the trials following a particular calibration. This was done so as to always have the most accurate gaze location tracking. Calibration was repeated when maximum error at validation was more than 1.33° of visual angle. Average validation error was always substantially lower than 1° of visual angle. The mean of the average validation errors was 0.35° of visual angle with a standard deviation of 0.09°. The mean of the maximum validation errors was 0.76° of visual angle with a standard deviation of 0.17°. To minimize head motion artifacts, all participants were seated on a stabilized drum stool with a back support, and had their heads fixed with a chin rest. Additionally, the “Head Camera” feature of the EyeLink II was engaged so as to provide some compensation for head motion that could still occur. Further, before each trial, a drift correction was performed.

### Stimuli

For the whole body stimuli, we utilized the same set of 18 grayscale male body image pairs as had been used in several recent studies of the body inversion effect [13–15,19]. This stimulus set had originally been produced with Poser 7.0 software to render bodies that had different physically plausible, yet non-meaningful, postures. The faces were also covered with a gray ellipsoid to prevent facial features from contributing to visual discrimination. Within each pair of images, the angle or position of an arm, a leg, and the head were altered slightly. At presentation, body images were centered on a white background and were scaled to have a height subtending approximately 10° of visual angle and a width subtending approximately 8° of visual angle, in conformity with the prior studies.

For the headless body stimuli, the whole body stimulus set was modified with Adobe Photoshop to remove the head from all images. Further, the inverted body stimuli were the same stimuli as the upright body stimuli, except vertically flipped about the same body-centric midline (i.e. near the pant line on each body, whether it was whole body or headless).

### Design and procedure

**Fig 1:**
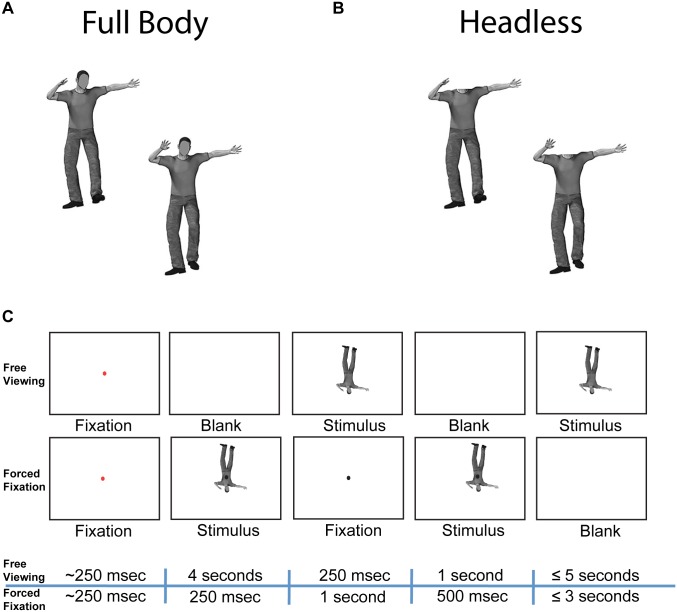
Stimuli and experimental paradigm. **(A)** Example pair of full body stimuli with different body postures. **(B)** Example pair of headless body stimuli with different body postures. **(C)** Trial sequences for Experiment 1 (Free Viewing) and Experiment 2 (Forced Fixation). During the forced fixation experiment, the fixation dot always appeared in the center of the screen, such that the designated body region (head, torso, or pelvis) was positioned at the fixation point.

Two experiments were conducted, and each participant took part first in Experiment 1 and then in Experiment 2. For both experiments, participants were required in each trial to respond whether two sequentially presented upright or inverted body images had the same or different body postures. In Experiment 1, participants were free to move their eyes while performing the task. In Experiment 2, however, maintenance of fixation on a particular body region (either head, torso, or pelvis on a given trial) was required while performing the task (Fig 1). The fixated body region differed pseudorandomly across trials.

Each of the two experiments had two sessions, one in which the task was performed on whole body stimuli, and one in which the task was performed on headless body stimuli. Each session was performed on different days, and the order of sessions was counterbalanced across participants. Within each session, upright bodies were displayed in half of all trials and inverted bodies were displayed in the other half; however, upright and inverted trials were pseudorandomly interleaved throughout each session.

For both experiments, a brief fixation point at the center of the screen was displayed at trial initiation. The remainder of the trial sequence did not begin until the participant fixated at the fixation point cumulatively for 250msec. If the participant had fixated away from the fixation point cumulatively for more than 1.5 seconds, a drift correction was then performed at the fixation point, and the trial initiation was attempted again.

#### Experiment 1 trial sequence

The trial sequence of Experiment 1 was designed to be directly comparable to the trial sequence in some prior studies of the body inversion effect [11,12,14,15,19]. Once a trial was initiated, a blank white screen was displayed for 4 seconds to allow participants to freely move their eyes in anticipation of the upcoming stimuli. Then the first body stimulus (250 msec duration) appeared in the center of the screen, followed by an inter-stimulus blank screen (1 second duration). A second stimulus, which was either the identical body image to the first image (same body posture trials) or was the image paired with the first stimulus (for details see “Stimuli” subsection above; different body posture trials), was then presented in the center of the screen. The second stimulus remained visible (up to 5 seconds) until the participant made a button-box response indicating whether the trial’s two body images had the same or different body posture. Participants were instructed to make a response even if unsure of the correct answer. The rarely occurring trials in which participants failed to make a response within 5 seconds were excluded from subsequent analysis. To avoid any influence of response laterality on the pattern of behavioral data, participants were required to make responses using both hands simultaneously. Specifically, button boxes were held in both hands and the two response options were designated with upper versus lower button responses. The reaction time was defined as the time until the first button was pressed, regardless of whether it was from the left or right hand. The mapping of body posture “same” versus “different” responses with the upper versus lower buttons was counterbalanced across subjects.

#### Experiment 2 trial sequence

The trial sequence of Experiment 2 was similar to that of Experiment 1, but modified so as to facilitate maintenance of fixation on the trial’s designated body region. Participants were required to maintain fixation on a visible fixation point at the center of the screen throughout the presentation of the stimuli and inter-stimulus blanks. Once a trial was initiated, the first body stimulus (250 msec duration) appeared such that the trial’s designated body region (head, torso, or pelvis) was positioned at the fixation point. The different body region conditions were thus simply vertical translations of one another. The inter-stimulus blank screen (1 second duration) then appeared with the central fixation point still visible. The second stimulus was then presented at the same screen position as the first stimulus. The second stimulus disappeared after 500msec, followed by a blank screen with no fixation point. The blank screen remained for up to 3 seconds until the participant made a button-box response indicating whether the two body images had the same or different body posture. The rarely occurring trials in which participants failed to make a response within 3 seconds were excluded from subsequent analysis (approx. 0.29% of trials on average) as were trials in which the eye-tracker detected deviation from the fixation point during the stimuli and/or inter-stimulus blank (approx. 13% of trials on average). Deviation from the fixation point was defined as any sample (besides during blinks) falling 2 or more degrees of visual angle in the vertical dimension and 1 or more degrees of visual angle in the horizontal dimension. These criteria were determined through a prior pilot study in which suitable thresholds were determined that were not overly sensitive to common incidental head motion artifacts on samples. As detailed in Methods in S1 Supplementary Materials, data from a prior pilot study of Experiment 1 determined the precise locations of the fixation points for the three designated body regions of Experiment 2. Participants were required to make responses using both hands simultaneously as in Experiment 1. Likewise, the mapping of body posture “same” versus “different” responses with the upper versus lower buttons was counterbalanced across subjects.

### Analyses

#### Software

Eye-movement and AOI data were obtained through EyeLink Data Viewer software by SR Research. Subsequent analyses on these data and behavioral data from the test phase were performed with custom Matlab (The MathWorks, Inc., Natick, MA, USA) code. Statistical tests were performed in SPSS (IBM, Somers, NY).

#### Behavior

We assessed participants’ discrimination performance, response bias, and reaction time on the old/new recognition task in the test phase. *d'* (*z*(hit rate)—*z*(false alarm rate)) and criterion *c* (-[*z*(hit rate) + *z*(false alarm rate)]/2) were computed for discrimination performance for each participant. Reaction times were analyzed for correct trials only. Reaction time analyses were performed on the medians calculated for each participant. Medians, rather than means, were calculated for each participant (as is common practice for reaction time analyses) because reaction time distributions tend to be skewed to high reaction times. The mean reaction times displayed in our figure are the means of the participant medians.

#### Eye-movement patterns

***Spatial density analyses***: Spatial density analyses, also known as “heat maps” (not presented), were used to derive the results of the profile density analyses that are presented from the data of Experiment 1. Spatial density analyses mapped the spatial density of eye-position samples on the screen during the presentation of the second body stimulus summed across trials as a function of our experimental manipulations. Eye-position samples during blinks and saccades were excluded from analysis. Each eye-position sample (one sample recorded every 4 msec) was plotted with equal density and spatial extent. Thus this analysis was equivalent to weighing the spatial density of each fixation by its duration. Specifically, individual samples were plotted as Gaussian densities with the peak density over the sample coordinate and a standard deviation of 0.31° of visual angle in both the x and y dimensions for each experimental session of each participant. Spatial density maps were simply summed within the same spatial reference frame across trials. They were summed without any spatial transformation since in Experiment 1 all body stimuli were aligned with one another across trials at the cores of the bodies (body extremities were not aligned, hence the suitability of the profile density analyses).

***Profile density analyses***: Because the positions of the body extremities were not aligned across trials, the spatial density heat maps described immediately above do not allow for adequate visualization of gaze over the extremities. To diminish these limitations, we calculated profile densities (i.e., densities summed along a single dimension of a heat map) during the presentations of the second stimulus in Experiment 1 for the different experimental conditions. The horizontal profile plots were produced by summing along the vertical dimension (y-axis) of a spatial density heat map, and vertical profile plots were produced by summing along the horizontal dimension (x-axis) of a spatial density heat map. The y-profile plots visualize gaze density over specific vertical features (e.g., head, torso, pelvis, legs) without respect to laterality or fine differences in horizontal position. The x-profile plots visualize the overall core versus lateral extremity gaze density. A group level profile density analysis was produced through summing the profile density analyses across participants.

***Profile density statistical contrast analyses***: Statistical tests were performed on contrasts between profile densities in order to map regions where significant differences in relative density were present between contrasted conditions. Because differences in relative density were of particular interest and because total amounts of eye-tracking data differed systematically among different conditions (e.g., longer reaction times for upright and inverted bodies meant there would be more density overall, even if relative density patterns were identical), profile densities were first normalized such that all trials had the same total density. Then a Monte-Carlo permutation test was conducted on these area-normalized profile density curves for each pair of contrasted conditions. Specifically, 360,000 resampling iterations were performed in which, under the null hypothesis of equivalence of profile density shapes between conditions, exchangeability of trials was assumed between the given contrasted conditions. In each iteration, trials’ profile densities were randomly assigned to the given subtrahend and minuend conditions being contrasted (equal numbers of trials in each condition), without regard to the actual condition associated with each trial. The two bins of data assigned as subtrahend and minuend profile densities were each summed together and the results were subtracted accordingly. These differences were then summed across participants. The differences in normalized profile densities between the contrasted conditions for the original data was compared to the iterations of permuted data at all given profile coordinate locations in order to determine the probability, under the null hypothesis, that differences as extreme or greater could be expected by chance. In this manner, two-tailed p-values were calculated to allow for mapping regions of statistically significant differences between curves.

Because there are many spatial coordinates for each profile density, a correction for multiple comparisons had to be completed. For each statistical profile density contrast, a false discovery rate (FDR) correction for multiple comparisons was performed. To conduct this correction, the p-values from the given statistical profile density contrast was passed to the Afni [27] function 3dFDR to produce q-values. Curves were plotted indicating a statistically significant differences at a threshold of q < 0.05, which corresponds to an estimated false discovery rate of 5% among the profile coordinates determined to be statistically significant.

The use of profile density statistical contrast analyses has been motivated in a prior eye-tracking study of face perception [28]. The present analysis is similar in principle to that in the prior study, but differs from it in a few details. Specifically, the prior study examined the profile density of the first five fixations and the permutation test involved exchanges for each ordinal fixation, whereas the current analysis examines normalized sample density and involves exchange of trials. Thus in the current analysis, fixation densities are effectively weighted by fixation duration and exchangeability is assumed for entire sequences of eye-movements (i.e., whole trials).

## Results

### Free viewing

#### Discrimination (*d’*) performance

We report a reduction in the body inversion effect for headless bodies. A two-way ANOVA on d’ discrimination scores from Experiment 1 (i.e., the free viewing experiment) with Headedness (whole body, headless) and Orientation (upright, inverted) as within-subject factors revealed a main effect of Headedness (*F*(1, 15) = 33.51, *p* < 0.0005, *η*_*p*_^*2*^ = 0.69) driven by higher discrimination performance for whole body trials and a main effect of Orientation (*F*(1, 15) = 49.84, *p* < 0.0005, *η*_*p*_^*2*^ = 0.77) driven by higher discrimination for upright trials. An interaction between Headedness and Orientation was also present (*F*(1, 15) = 10.47, *p* < 0.007, *η*_*p*_^*2*^ = 0.41).

**Fig 2:**
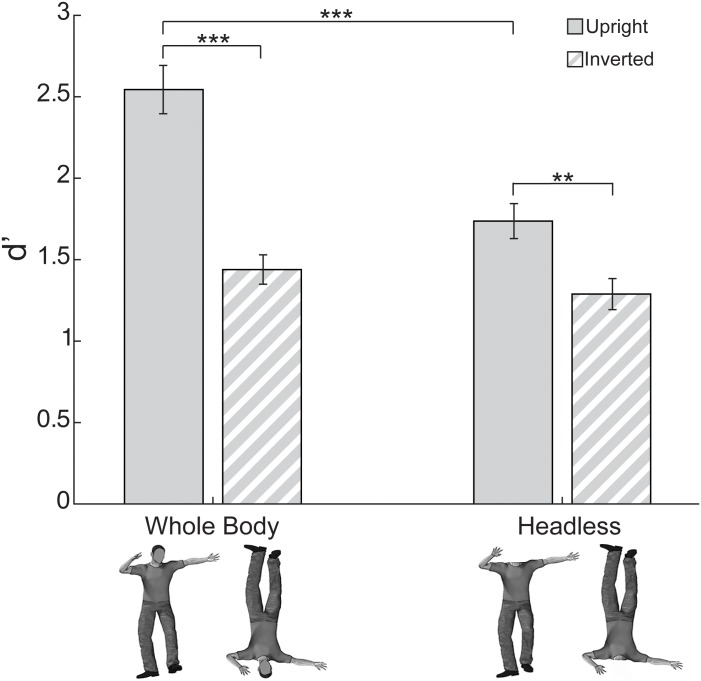
Discrimination performance (d’) in Experiment 1 (free viewing). The BIE was smaller for headless bodies compared to whole bodies. This was driven by lower discrimination performance for upright headless bodies compared to upright whole bodies (** = p < 0.01, *** = p < 0.001). Error bars indicate standard error of the mean.

Planned t-tests investigating the interaction between Headedness and Orientation (Fig 2) revealed that it was driven by a reduction in performance for upright headless trials compared to upright whole-body trials (paired *t*(15) = 6.61, *p* < 0.0005 two-tailed, bias corrected *G*_*Hedges*_ = 1.47). Inverted headless trials did not differ significantly from inverted whole-body trials (paired *t*(15) = 1.08, *p* > 0.29 two-tailed, bias corrected *G*_*Hedges*_ = 0.38), suggesting that discrimination performance was equivalent for inverted trials regardless of whether the body was whole or headless. A BIE was detected for whole body trials (paired *t*(15) = 6.09, *p* < 0.0005 two-tailed, bias corrected *G*_*Hedges*_ = 2.12). Notably, despite lower upright body discrimination performance, an inversion effect was also present for headless body trials (paired *t*(15) = 4.11, *p* < 0.002 two-tailed, bias corrected *G*_*Hedges*_ = 1.04).

#### Response bias (Criterion *c*) and reaction time

Response bias (Fig C in S1 Supplementary Materials) was more conservative (i.e. lower tendency to respond “different posture” when in doubt) for upright headless trials than for the other conditions in Experiment 1. Reaction time (Fig D in S1 Supplementary Materials) was lower for upright than inverted body trials in Experiment 1. Full statistics for response bias and reaction time in Experiment 1 are available in Results in S1 Supplementary Materials.

#### Distribution of gaze

We analyzed gaze patterns separately for the first and second body stimuli of the experimental trials. Our participants’ gaze patterns during the first stimuli (Figs O-T in S1 Supplementary Materials), reveal fairly focal gaze density at the screen-centric upper half of the body stimuli. Since few, if any, saccades would have been possible during the brief (250 msec) stimulus duration, this finding also indicates that participants preemptively placed their gaze at this location before the first stimulus appeared. Because the brief duration of the first stimulus limited saccades, the primary focus of our analysis is on the gaze patterns during the second stimulus, which are described in detail in the following paragraphs.

Overall, the analyses of gaze during the second body stimuli indicate that inversion of bodies shifted participants’ gaze away from the upper body predominantly to the pelvis area. Further, in the case of upright bodies, headlessness appears to strongly shift gaze away from the head region to the torso region.

**Fig 3:**
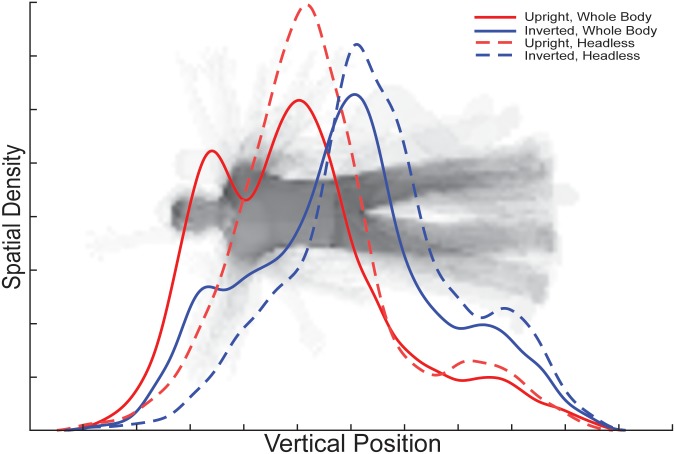
Vertical profile densities. The curves visualize relative eye-movement densities over specific vertical positions along the body during the presentation of the second body stimuli and represent the spatial densities of eye-movements summed along the horizontal dimension for each condition.

**Fig 4:**
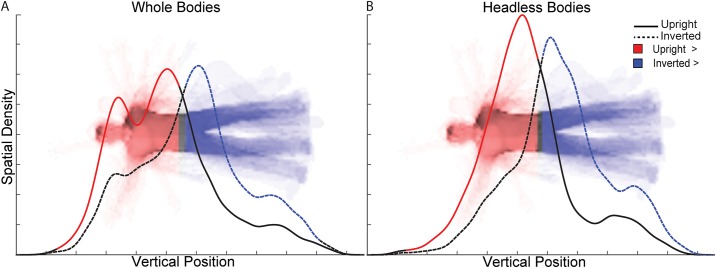
Vertical profile density statistical contrasts between upright and inverted body trials. The curves in separate plots for **(A)** whole and **(B)** headless body trials represent the vertical profile densities during the presentation of the second body stimuli for the upright and inverted body trials. For reference, the head is displayed in the headless bodies plot, even though it was absent in the actual stimuli. Vertical positions at which upright body density was statistically significantly greater (*q* < 0.05) than inverted body density are indicated with red shading of the curve and image. The inverse is indicated in blue.

**Fig 5:**
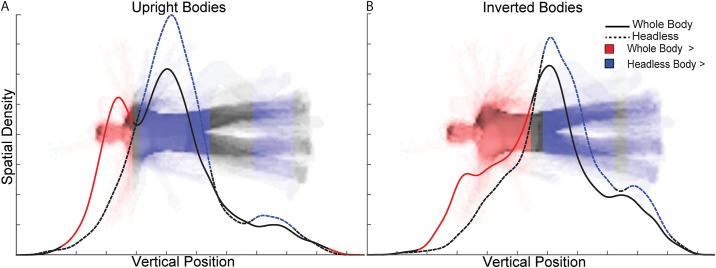
Vertical profile density statistical contrasts between whole and headless body trials. The curves in separate plots for **(A)** upright and **(B)** inverted body trials represent the vertical profile densities during the presentation of the second body stimuli for the whole and headless body trials. Vertical positions at which whole body density was statistically significantly greater (*q* < 0.05) than headless body density are indicated with red shading of the curve and image. The inverse is indicated in blue.

Analyses of the profile densities of gaze along the y-axis (Figs 3–5) indicate that the different Orientation and Headedness conditions modulated gaze profile density patterns, and therefore modulated the body features gazed upon. For upright whole body trials, the profile density of gaze along the y-axis indicates group-level peaks at the torso and head regions of comparable relative density, but with torso being numerically slightly higher in density. However, for inverted whole body trials, the gaze density clearly shifted away from the head and torso to predominantly the pelvis area. Overall, participants gazed toward the upper body significantly more for upright than inverted bodies and toward the lower body significantly more for inverted than upright bodies (Fig 4).

For upright headless body trials, there was a strong peak in profile density over the torso region, but no longer over the head region (i.e., where the head would be). This difference was confirmed as significant in the statistical analyses, along with a small relative increase in gaze to the legs for upright headless bodies (Fig 5A). Inverted headless bodies received peak profile density over the pelvis, similarly to inverted whole bodies. Nonetheless, moderate differences in the profile densities between inverted whole and headless bodies were significant, indicating that the upper body was gazed at relatively more for whole inverted bodies, and the lower body was gazed at relatively more for inverted headless bodies (Fig 5B).

In the x-dimension, participants’ gaze predominantly landed near the midline of the body, albeit with rather slight lateral differences in density between conditions, as detailed in S1 Supplementary Materials.

### Forced fixation

#### Discrimination (*d’*) performance

**Fig 6:**
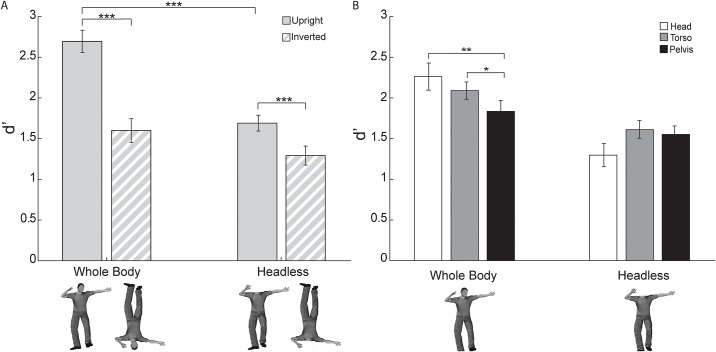
Discrimination performance (d’) in Experiment 2 (forced fixation). These two analyses investigated the independent two-way interactions of orientation and headedness and of fixation location and headedness. **(A)** The BIE was again reduced for headless compared to whole bodies due to a reduction of discrimination performance for headless upright compared to whole upright bodies. The three fixation location conditions were pooled together in this analysis (*** = p < 0.001). **(B)** For whole body trials, discrimination performance was relatively lower for pelvis than head and torso, but for headless body trials, discrimination performance did not differ among fixation locations. Upright and inverted conditions were pooled together for this analysis (* = p < 0.05, ** = p < 0.01). For both plots, error bars indicate standard error of the mean.

In Experiment 2, there was overall higher discrimination performance for whole than headless bodies and for upright than inverted bodies. An interaction between Headedness and Orientation (Fig 6A) conceptually replicated the reduction in inversion effect for headless body trials compared to whole body trials as seen in Experiment 1 and in the prior studies of the body inversion effect [13–15,19]. Importantly, an interaction between Headedness and Location (i.e., fixation at head, torso, or pelvis) indicated that the effect of Location differed by Headedness (Fig 6B). Specifically, for whole body trials, discrimination performance was relatively lower for pelvis than head and torso, but for headless body trials, discrimination performance did not differ among the fixation locations. Taken together, these results provide evidence that for whole body trials, influences of feature visibility (specifically of the head) and of configural (typical versus atypical orientation) processing differences drive the inversion effect, whereas for headless body trials, configural processing differences exclusively drive the inversion effect. The absence of any significant interactions involving both Location and Orientation suggests that fixating at different locations did not modulate the magnitude of the BIE (i.e., the difference in performance between upright and inverted body conditions) for either whole bodies or headless bodies.

A three-way ANOVA on d’ discrimination performance from Experiment 2 with Headedness (whole body, headless), Orientation (upright, inverted), and Location (head, torso, pelvis) as within-subject factors revealed main effects of Headedness (*F*(1, 15) = 30.96, *p* < 0.0005, *η*_*p*_^*2*^ = 0.67) and Orientation (*F*(1, 15) = 88.10, *p* < 0.0005, *η*_*p*_^*2*^ = 0.86) driven by higher discrimination performance for whole than headless bodies and for upright than inverted bodies. No main effect of Location was detected (*F*(1.79, 26.79) = 1.25, *p* > 0.29 Greenhouse-Geisser corrected, *η*_*p*_^*2*^ = 0.077). A two-way interaction of Headedness and Orientation (*F*(1, 15) = 15.94, *p* < 0.0015, *η*_*p*_^*2*^ = 0.52; Fig 6A) was driven by the same effects (detailed below) as those seen in the interaction of Headedness and Orientation in the discrimination performance data from Experiment 1. Importantly, a two-way interaction between Headedness and Location was present (*F*(1.79, 26.92) = 9.15, *p* < 0.002 Greenhouse-Geisser corrected, *η*_*p*_^*2*^ = 0.38; Fig 6B) indicating, as hypothesized, that the influence of Location varied by Headedness. No two-way interaction existed between Orientation and Location (*F*(1.27, 19.03) = 0.63, *p* > 0.47, *η*_*p*_^*2*^ = 0.040). There was also no three-way interaction among Headedness, Orientation, and Location (*F*(1.88, 28.14) = 0.099, *p* > 0.89, *η*_*p*_^*2*^ = 0.007; Fig U in S1 Supplementary Materials). A lack of any significant two- or three-way interaction involving both Orientation and Location suggests that fixation at different locations did not modulate the magnitude of the BIE (i.e., the difference in performance between upright and inverted body conditions; Fig V in S1 Supplementary Materials), although there was a main effect of Orientation that modulated absolute discrimination performance. The lack of the three-way interaction further indicates that the two significant two-way interactions existed independently of the other respective factors, which critically thus allowed us to investigate those two two-way interactions separately.

The two-way interaction between Headedness and Orientation in Experiment 2 (Fig 6A) conceptually replicated the same interaction as was seen in Experiment 1. A series of planned t-tests on discrimination performance analyzed by Headedness and by Orientation (Location pooled) revealed that the interaction was again driven by a reduction in performance for upright headless trials compared to upright whole-body trials (paired *t*(15) = 8.16, *p* < 0.0005 two-tailed, bias corrected *G*_*Hedges*_ = 1.99), indicating the importance of the head posture information for the discrimination task. Inverted headless trials did not differ significantly from inverted whole-body trials (paired *t*(15) = 1.93, *p* > 0.071 two-tailed, bias corrected *G*_*Hedges*_ = 0.55), though the difference was marginally significant, suggesting that discrimination performance for inverted headless body trials was equivalent to or possibly somewhat lower than that of inverted whole body trials. A body inversion effect (i.e. greater upright than inverted body posture discrimination performance) was detected again for whole body trials (paired *t*(15) = 8.99, *p* < 0.001 two-tailed, bias corrected *G*_*Hedges*_ = 1.83). Notably, again despite lower upright body discrimination performance, an inversion effect was also present for headless body trials (paired *t*(15) = 3.62, *p* < 0.0025 two-tailed, bias corrected *G*_*Hedges*_ = 0.87).

The two-way interaction between Headedness and Location (Fig 6B) indicated that the effect of Location differed by Headedness. Specifically, for whole body trials, discrimination performance was relatively lower for when fixating at the pelvis than at the head and at the torso, but for headless body trials, discrimination performance was relatively lower for the head region than torso and pelvis, as revealed in the following series of t-tests investigating this interaction. For whole bodies, discrimination performance at the pelvis was significantly lower than at the head (paired *t*(15) = 3.20, *p* < 0.0065 two-tailed, bias corrected *G*_*Hedges*_ = 0.66) and torso (paired *t*(15) = 2.53, *p* < 0.024 two-tailed, bias corrected *G*_*Hedges*_ = 0.49); however, performance did not significantly differ between head and torso fixation (paired *t*(15) = 1.35, *p* > 0.19 two-tailed, bias corrected *G*_*Hedges*_ = 0.29) for whole bodies. For headless bodies, discrimination performance at the head region did not differ significantly from that at the torso (paired *t*(15) = 2.08, *p* > 0.054 two-tailed, bias corrected *G*_*Hedges*_ = 0.58) or pelvis (paired *t*(15) = 1.70, *p* > 0.10 two-tailed, bias corrected *G*_*Hedges*_ = 0.48), though the lower performance at the head region than the torso approached significance. Importantly, discrimination performance also did not differ between torso and pelvis fixation (paired *t*(15) = 0.48, *p* > 0.64 two-tailed, bias corrected *G*_*Hedges*_ = 0.13) for headless bodies, and so the potential hypothesis that the inversion effect for headless bodies during free-viewing is driven, even partially, by preferred gaze at the torso versus the pelvis for upright and inverted trials, respectively, is not supported. Thus, given this null influence of differential fixation location, the alternative hypothesis that the atypical configuration (inversion) *per se* alone drives the inversion effect for headless bodies could not be rejected. Lastly, with respect to discrimination performance differences between whole and headless bodies at each location, discrimination scores for headless bodies were significantly lower at the head (t(15) = 6.01, p < 0.0010, two-tailed, bias corrected *G*_*Hedges*_ = 1.47) and torso (t(15) = 4.01, p < 0.0012, two-tailed, bias corrected *G*_*Hedges*_ = 1.05), and marginally lower at the pelvis (t(15) = 1.90, p = 0.077, two-tailed, bias corrected *G*_*Hedges*_ = 0.56).

#### Response bias (Criterion *c*) and reaction time

Response bias differed independently by Headedness and by Location (Fig W in S1 Supplementary Materials). Specifically, there was a lower (i.e., more liberal) criterion for responding “different posture” for whole than headless bodies and a lower criterion for head-region-focused trials than for torso-focused trials. Also, for upright headless trials and for pelvis-focused inverted headless trials, participants applied criteria that were more conservative than optimal. The conservative criteria for upright headless trials in Experiment 2 conceptually replicate the conservative criterion for upright headless trials observed in Experiment 1. Also in Experiment 2, Orientation and Headedness independently influenced reaction times such that reaction time was lower for upright than inverted body trials, and lower for whole than headless body trials (Fig X in S1 Supplementary Materials). Full statistics for response bias and reaction time in Experiment 2 are available in Results in S1 Supplementary Materials.

## Discussion

We report differential gaze patterns to upright compared to inverted bodies within an experimental paradigm that is highly comparable to prior studies of the BIE. In particular, our participants predominantly gazed at the (body-centric) upper body for upright bodies, and at the lower body for inverted bodies. Given the informativeness of head posture for the body posture discrimination task, this specific pattern of gaze preferences contributed to the BIE for whole bodies as revealed in subsequent controlled manipulation of fixation location. This manipulation revealed that discrimination performance for whole bodies when fixating at the upper body (head or torso) was relatively higher than when fixating at the lower body (pelvis), regardless of whether the body was upright or inverted. In addition to this influence of the differential gaze patterns, seeing a body in a typical versus an atypical configuration as such (i.e. upright versus inverted orientation *per se*) also drives the existence of the BIE. Specifically, we found that a BIE could be detected for both whole and headless bodies even when fixation location on the body was held constant, though inclusion of the head posture information was still highly discriminative in the context of such configural processing (i.e., processing relating to the feature arrangement information “more than the sum of its parts”). Interestingly, the influence of differential configuration to the BIE appears greater than that of the differential preferred gaze locations.

### The BIE and its reduction for headless bodies

The results of Experiment 1 are consistent with prior studies of the BIE that reported a reduction in the effect for headless compared to whole bodies, driven by a reduction in discrimination performance (d’) for upright headless compared to upright whole bodies. Except for measurement of eye-movements during the experiment, the stimuli and experimental paradigm of Experiment 1 were identical to those found in three prior studies of the BIE [14,15,19] and similar to those in a fourth study [13]. Thus our results can be compared most especially to those of these prior studies.

Four prior studies [13,14,16,19] suggest that a reduction in the BIE led to a complete abolishment of the BIE for headless bodies, whereas two other prior studies indicate that the reduction did not lead to a total abolishment of the BIE [15,29]. Consistently with the latter studies, we also detected a significant BIE for headless bodies, albeit of reduced magnitude than that observed for whole bodies. We found that the effect size of the BIE for headless bodies (bias corrected *G*_*Hedges*_ = 1.04) was about half that for whole bodies (bias corrected *G*_*Hedges*_ = 2.12); therefore, the failure of some prior studies to detect the BIE for headless bodies is likely due to relatively lower statistical sensitivity in those studies. Differences in statistical sensitivity for the headless BIE could have been influenced by any relevant factors that varied across studies, such as the number of participants in the analysis (10 to 40 participants), number of headless body trials (16 to 240 trials), performance measure (accuracy versus d’), task (same/different, match to sample, study/test), stimulus display time, or stimulus set.

### Orientation-specific gaze patterns

For whole bodies, participants gazed most at the torso and head of upright bodies, whereas they predominantly gazed at the pelvis for inverted bodies. For headless bodies, participants’ gazed predominantly at the torso of upright bodies, and at the pelvis of inverted bodies. Our gaze pattern results are consistent with the relative pattern of fixations between upright and inverted whole bodies reported in the only prior study of the BIE to include measurement of eye-movements [18]. The absolute fixation patterns for inverted whole bodies in that study seems to differ from our gaze results though, likely because of differences in body stimulus design. Unlike our study, that study did not measure eye-movements to headless bodies and, importantly, did not subsequently investigate the functional significance of the observed eye-movements.

A prior study reported that when stimulus head posture was held constant, the BIE was reduced, resembling the abolishment of the BIE for headless bodies; however, removing the arm or leg posture information did not reduce the BIE to the same degree [14]. Thus, that study of Yovel and colleagues provided evidence that the head posture information is the most important factor for discrimination of body posture. Given the importance of the head posture information in the task and given that our participants tended to gaze at or nearer to the head region for upright bodies compared to inverted bodies, it seems likely then that the specific preferential patterns of gaze that we observed influenced the BIE for the whole bodies. Since the BIE was reduced for headless bodies, it also seems likely that the differential gaze patterns could not strongly, if at all, drive the BIE similarly for the headless bodies, given that the head posture information was absent.

The difficulty in directly inferring visual information use from spatial gaze patterns alone has been indicated in prior eye-tracking studies [30–32]. However, the peak in gaze density at the torso for upright bodies likely enabled sufficient utilization of the head information of whole bodies, even when there was no direct gaze to the head (see Head AOI Analyses in Results in S1 Supplementary Materials). That peak gaze density was found at the pelvis of inverted bodies for both whole and headless bodies and that discrimination performance was as low for inverted whole as for inverted headless bodies also suggest, though, that gazing sufficiently far from the head did significantly impair, and possibly even entirely precluded, utilization of the head posture information in whole bodies. The results of Experiment 1 are, thus, strongly suggestive of a relationship between differential preferred gaze patterns and the BIE, which Experiment 2 confirms.

### Differences in fixation location causally modulated discrimination performance for whole bodies

From the results of our subsequent forced fixation experiment (i.e., Experiment 2), we can infer that these differential preferred gaze patterns did indeed functionally contribute to the free viewing BIE for whole bodies. The fixation location (i.e., head, torso, or pelvis) manipulation of Experiment 2 modulated body posture discrimination performance; however, the modulation depended on whether bodies were whole or headless. Specifically, for whole body trials, discrimination performance was relatively lower for pelvis than head and torso, but for headless body trials, discrimination performance did not differ among different fixation locations. This modulation in discrimination performance by fixation location and headedness was statistically independent of the modulation by body orientation, and so reveals the pattern of *relative* discriminative advantages of fixating at these different body locations on whole and headless bodies, regardless of whether the body is upright or inverted. Because fixation at the pelvis caused relatively poorer discrimination than fixation at the torso and head for whole bodies, it is reasonable to conclude that the tendency during free viewing (i.e., Experiment 1) to gaze at the pelvis for inverted whole bodies and the torso and head of upright whole bodies at least partially drives the BIE for whole bodies during free viewing.

By similar reasoning, the lack of modulation in discrimination performance between torso and pelvis fixation for headless bodies reveals, at least in part, why the similar tendency during free viewing to gaze at the torso for upright headless bodies and the pelvis for inverted headless bodies does not lead to a BIE nearly as pronounced as that for whole bodies during free viewing. That we detected a significant BIE for headless bodies in Experiment 1, although of smaller magnitude than for whole bodies, interestingly suggests though that some other orientation-specific processing differences besides differences in gaze pattern drives the BIE for headless bodies. From this it is reasonable to hypothesize that such configural processing differences may also partially drive the BIE in whole bodies.

The hypothesis that the observed pattern of relative discriminative saliences among the body parts could be explained as trivially reflecting the low-level image properties of our chosen stimulus set can be rejected, since the observed discriminative advantage for the upper body locations (head and torso) of whole bodies does not correspond to the image region containing by far the greatest number of pixel differences between our stimulus pairs, namely the legs (Results and Fig Z in S1 Supplementary Materials). On the contrary, if the salience of body part posture changes were driven merely by the low-level properties of our stimuli, we should have instead observed that discrimination performance was highest at the pelvis fixation location since it is closest to the legs. Further the removal of the head from the stimuli would not have been so detrimental to discrimination performance, since the head region had the least degree of low-level image differences between stimulus pairs. Thus, the pattern of relative discriminative advantages that we observed among body parts is not a mere artefact of our chose stimulus set, but rather reflects a general pattern of salience driven by some high-level neurocognitive mechanism.

### Orientation-specific configural processing also influenced the BIE

We found evidence for configural processing differences between upright and inverted bodies for both whole and headless bodies in the results of Experiment 2, suggesting that during free viewing these configural processing differences drive the BIE for headless bodies, and largely drive the BIE for whole bodies. We found an interaction between the effects of body orientation and headedness (*p* < 0.0015, *η*_*p*_^*2*^ = 0.52) in discrimination performance in Experiment 2 that highly resembled the interaction found in Experiment 1. Specifically, a BIE was detected for whole body trials but, again, despite lower upright body discrimination performance, an inversion effect was also present for headless body trials (Fig 6A). This interaction between body orientation and headedness was not modulated by fixation location (i.e., there was no three-way interaction among body orientation, headedness, and fixation location: p > 0.89) and so importantly reveals that the BIE and its modulation by headedness did not depend on the fixation location (Figs U and V in S1 Supplementary Materials). These results for discrimination performance provide evidence that in the context of free viewing, beyond any influence of differences in preferred gaze pattern, an independent influence of the body orientation as such on processing also exists. Such an influence of body orientation *per se* in body processing can also be seen in the reaction time results of Experiment 2, in which reaction time was longer for inverted than upright bodies. This influence of body orientation on reaction time was independent of the increased reaction time for headless compared to whole bodies. Taken together, these results indicate that configural processing is different between upright and inverted body stimuli, although inclusion of the head posture information is highly discriminative in the context of such configural processing.

### Relative contributions of differential gaze location and differential configural processing to the BIE

The results of Experiment 2 indicate that for whole body trials, influences of feature visibility (specifically of the head) and of configural processing (of typical versus atypical orientation *per se*) differences together drive the BIE during free viewing, whereas for headless body trials, only configural processing differences seem to drive the inversion effect. Interestingly, these results also contain evidence of the *relative* contributions of feature and configural factors to the free viewing BIE. This is because the influence of feature and configural factors could be investigated independently in Experiment 2 for both whole and headless bodies given that fixation location and body orientation each independently interacted with headedness in their influences on discrimination performance.

In the case of headless bodies, it is obvious that configural processing differences between the upright and inverted orientations overwhelmingly drove the BIE during free viewing. The non-significant difference in discrimination performance between torso and pelvis fixation in Experiment 2 plainly reveals that the tendency to prefer to gaze at the torso of upright headless bodies and to gaze at the pelvis of inverted headless bodies during free viewing (as reported in Experiment 1) plays a negligible if, any role in the free-viewing BIE for headless bodies.

Less obvious is the relative contribution of feature and configural processing in the BIE for whole bodies during free viewing, though our data from Experiment 2 allow for an estimate that suggests that the impact of body orientation *per se* has over four times the impact on discrimination performance than the difference in preferred gaze location between upright and inverted whole bodies during free viewing has. The difference in d’ between torso and pelvis fixation for whole bodies (orientation pooled) in Experiment 2 is approximately 0.25, quantifying the magnitude of the influence that such differential fixation has on discrimination performance. The difference in d’ between upright and inverted whole bodies (fixation location pooled) in Experiment 2 is approximately 1.10, thus quantifying the magnitude of the independent influence that body orientation as such has on discrimination performance. Thus, the influence of body orientation *per se* on discrimination performance is greater in magnitude than that of the differential gaze patterns.

Several other studies are consistent with our results suggesting distinct feature and configural processing for bodies. Indeed the neural substrates of such perceptual processes are beginning to be uncovered. One study reports a gradual increase in selectivity of response in the extrastriate body area (EBA) as a function of the amount of body shown, but a step-like function in the selectivity of the fusiform body area (FBA) that also showed no significant selectivity for individual fingers or hands [33]. Thus, it was concluded that the EBA processes bodies at the level of parts, and the FBA processes body configural information. Our results suggest that preferential gaze patterns would drive differential activation in the EBA and that body orientation as such would drive differential activation in the FBA.

### What is the nature of the configural processing differences driving the BIE?

The apparent relative importance of such configural processing in the BIE opens up questions as to what the nature and mechanism of such processing may be. Further, whether the mechanism of such configural processing is specific to bodies, or rather, whether these processes are general to other categories of stimuli is unclear. We propose a few possibilities for mechanisms of the configural processing differences that underlie the BIE. Such possibilities are not mutually exclusive.

One possible mechanism for the decrease in discrimination performance for inverted bodies is that of violated expectations that leads to a misallocation of spatial attention to inverted bodies. Because bodies are typically experienced in an upright orientation and, in the context of the experiments of the current study, the orientation of the upcoming stimuli are unknown, perhaps participants tend, by default, to employ attention optimized for discriminating upright bodies, until an inverted body is perceived. Thus when an inverted body trial occurs, participants are not as well prepared to process the stimulus as they tend to be for upright body stimuli. Indeed, if fixation on the upper half of the body is part of the default strategy for processing upright bodies, some misallocation of attention due to violated expectations may partially explain why our participants tended to consistently direct eye-movements to the screen-centric upper half of the body stimuli, even when they were inverted. Analysis of our participants’ gaze patterns during the first stimulus (Figs O-T in S1 Supplementary Materials), during which few, if any, saccades would have been possible due to the brief (250 msec) stimulus duration, reveals that participants did indeed preemptively place their gaze at the screen-centric upper half of the upcoming body stimuli, consistent with an expectation for upright bodies across trials. Gaze patterns during the second stimulus were similar in that the screen-centric upper stimulus bias persisted, suggesting that whatever determined the preemptive placement of the eyes before the start of the trial is a key contributory factor to the BIE in the context of the posture change discrimination task. A future study in which expectation of stimulus orientation is manipulated could test this hypothesis of misallocation of attention due to violated expectations. It is worth noting however, that since misallocation of attention to inverted stimuli could be expected to be a general phenomenon, and not specific to bodies or faces, it might be expected that a comparably sized inversion effect would be observed for other visual stimuli. However, the inversion effect for houses and other (non-face) stimuli have been reported as being smaller in magnitude than the BIE [11,12,16]. Further, a prior study reports a BIE even when participants could fully expect the body orientation because upright and inverted conditions were segregated into separate experimental blocks [29].

Another possibility is that through basic visual perceptual learning [34–39], bodies are better processed when their features are seen in the most commonly experienced configuration (orientation) and when they fall at the most commonly experienced retinal location. In such a case, the relevant mechanism for the BIE would consist in differential sensitivity to low-level visual features of body stimuli among different visual receptive fields. The specific patterns of visual field biases for the EBA and FBA may reflect this [40,41]. Evidence suggestive of this possibility comes from a prior study from two of our authors [42] in which body part discrimination performance and fMRI pattern discriminability in the right EBA were highest for stimuli in their typical combinations of visual field and body side (e.g. right visual field, left body part). Further, another study [12] reported an inversion effect for intact bodies, but not for stimuli in which the location of specific body parts have been unnaturally permuted. Indeed, discrimination performance for those scrambled bodies were comparable to that for the inverted intact bodies.

A third possibility is that, in a manner highly analogous to what has been proposed for faces [10,43–45], bodies are processed through specialized holistic visual perceptual mechanisms, through which the complex relationships among individual features of a given body are integrated to form a unitized representation. Either because of innate visual processing mechanisms predisposed for visual body processing or because of the visual expertise gained through extensive visual experience with bodies, such possible holistic processing mechanisms, specialized for upright bodies, may explain the configural processing differences driving the BIE and the relatively reduced BIE for headless bodies.

Evidence of at least some sort of specialized visual processing for bodies along these lines comes from the existence of cortical regions that respond selectively to visual body stimuli [1–5]. This evidence is strengthened by a study [46] that revealed that the transcranial magnetic stimulation (TMS) applied to the right EBA disrupts visual discrimination of bodies, but not of faces or objects. A double dissociation was further reported such that visual discrimination of faces, but not of bodies or objects was disrupted with TMS applied to the right occipital face area (OFA), a face-selective cortical region, suggesting that body visual processing does not rely on face processing mechanisms. Similarly, discrimination of objects, but not of bodies or faces was disrupted by TMS applied to the right lateral occipital area (LO), an object-selective cortical region, suggesting that body processing is truly specialized as it does not rely on other general object processing mechanisms.

There is even evidence that body-selective brain regions are implicated in some manner of configural visual processing of bodies and that body inversion disrupts such processing. For example, a neuroimaging study [47] involving multivoxel pattern analysis (MVPA) reports that differences in MVPA discrimination for whole bodies compared to disjointed body parts was reduced for inverted versus upright bodies in the body-selective brain regions EBA and FBA. In that study, MVPA discrimination in the body-selective areas was stronger for whole compared to disjoint body parts, regardless of whether the regions had been functionally defined by selectivity to whole bodies, headless bodies, or disjoint body parts, suggesting that configural processing is general to occipital-temporal body-selective areas. Further evidence for configural processing in at least the right FBA is also reported in another neuroimaging study [48] showing selective adaption to whole bodies compared to disjoint body parts. The existence of specialized visual processing for bodies does not necessarily imply that the configural processing of bodies is holistic though.

The existing evidence relating to configural visual processing of bodies [11–14,33,47–56] has been variously interpreted with respect to whether configural body processing is truly holistic. In large part, this may be because what constitutes holistic processing has no clear consensus within the cognitive science community at large. One representative study reported an inversion effect both for whole bodies and for vertically split half bodies, but not for individual body parts, for unnaturally reconfigured bodies, or for horizontally split half bodies [12]. While this was interpreted as indicating that the BIE is not dependent on comparisons between the fully unitized non-decomposable representations possible only for whole bodies, it was nonetheless concluded that bodies are, in some sense, processed in a holistic manner. Given that inversion effects were also observed for vertically split half face stimuli, and that faces are widely regarded as processed holistically, the full set of results could not preclude that bodies are processed holistically. However, in a later study [14] inversion effects were observed for bodies missing arms or legs, but not for whole bodies in which the head posture was held constant or for headless bodies. From this it was concluded that the head posture information played a strong role in the discrimination of bodies, but has also been interpreted [13] as refuting the hypothesis that bodies are processed holistically, because bodies did not need to be complete for a BIE to be observed. Further study and a clearer definition of holistic processing are required to resolve this question.

A final possibility is that face-processing mechanisms drive the BIE, given the disproportionate importance of the head posture information for the discriminability of body posture. Evidence consistent with this comes from an fMRI study [13] investigating neural adaptation within body-selective, face-selective, and object-general cortical visual processing regions during a task similar to the one in the present study and with identical stimuli. Mirroring the behavioral BIE observed in that study for whole, but not for headless bodies, the face-selective fusiform face area (FFA) showed an adaptation effect to upright but not to inverted bodies, but only for whole bodies. Body-selective areas, on the other hand, exhibited similar adaptation effects for upright and inverted whole and headless bodies, suggesting that discrimination in these regions was similar for the two orientations regardless of the presence of the head. Object-general areas showed no adaptation to body posture for any of the conditions. Thus, only the adaptation pattern in the FFA was consistent with the BIE seen in their behavioral results for whole but not for headless bodies.

We note however, that our findings challenge the given interpretation of that fMRI study since the results could be explained by the differential pattern of gaze observed in our study. Specifically, for whole bodies, a tendency to gaze at or near the head could have driven the adaptation effect reported for upright whole bodies in the FFA, whereas the tendency to gaze far away from the head at the pelvis for inverted bodies could have induced the abolishment of the adaptation. Headless bodies, since they lack any head posture information would similarly not be expected to induce adaptation effects within the FFA. Indeed, in our study, gaze patterns as well as discrimination performance for inverted bodies were comparable between whole and headless bodies. The additional results in the fMRI study indicating a similar magnitude of adaptation in body-selective regions for whole and headless bodies can also be interpreted as consistent with our result of a significant BIE in behavior for both whole and headless bodies. In the fMRI study, though inverted bodies elicited a similar magnitude of adaptation in body-selective regions as upright bodies, they also elicited overall greater magnitude BOLD responses in those same areas, compared to upright bodies in the case of both whole and headless bodies. This increased BOLD response could be interpreted as indicating a reduced neural efficiency for processing inverted bodies. If this is true, then the BIE we observed for both whole and headless bodies may thus be partially explained by reduced neural efficiency in the EBA and FBA for processing inverted bodies. Such an account is consistent with the results of a TMS study that reports that TMS applied to the EBA selectively impaired discrimination of inverted bodies [54], but did not affect discrimination of upright bodies (though it has been reported that TMS to EBA can disrupt upright body discrimination in Pitcher et al., 2009), suggesting that representations of bodies in the EBA are more stable, and thus more resistant to TMS disruption, for upright than inverted bodies. Alternatively, if the increased BOLD response in the EBA and FBA to inverted bodies actually rather reflects greater sensitivity to inverted bodies, then the reported reduced response to inverted bodies in body-selective regions in posterior temporal cortex [55,56] might indicate other body-selective regions implicated in the processing mechanisms driving the BIE.

Further, a study on acquired prosopagnosic patients suggests that impairment in face identification mechanisms do not impact body processing [15]. Specifically, the face inversion effect, but not the BIE, was reduced in the patients compared to controls. Another study suggests, however, that, if not face identification mechanisms, face detection mechanisms may be involved in the BIE [19]. The study specifically measured the magnitude of the inversion effect for faces, isolated faceless head stimuli, and faceless heads in a full or partial body context. The faceless stimuli that were associated with a robust inversion effect, namely the faceless heads in full and partial body contexts, were also associated with a greater likelihood to spuriously rate confidence in detection of a face in a separate task. Thus, even if face identification mechanisms are not specifically employed during the processing of bodies, it is possible that early perceived detection of a face due to contextual cues associated with the presence of a face may engage other face perception mechanisms or may otherwise condition subsequent processing of bodies in such a way as to produce the BIE.

## Conclusion

The present study uncovered human perceptual mechanisms underlying visual discrimination of bodies. Consistent with prior studies, we observed the BIE and its reduction for headless bodies compared to whole bodies. Importantly, we provide causal evidence that differences in feature visibility brought about by differential gaze patterns and that configural processing differences associated with the orientation of bodies both independently contribute to the BIE and its reduction for headless bodies. Further research is required to elucidate the neural substrates and specific mechanisms of these processes.

## Supporting Information

S1 Supplementary Materials Supplementary Methods, Results, and Figures. (DOCX)
