# Supplementary material for: Where You Look Matters for Body Perception: Preferred Gaze Location Contributes to the Body Inversion Effect
Source: PLoS One. 2017 Jan 13;12(1):e0169148. doi: 10.1371/journal.pone.0169148 (PMC5234795; doi:10.1371/journal.pone.0169148)
Supplement: S1 Supplementary Materials — (DOCX) [file pone.0169148.s001.docx]

**Supplementary Materials**

**Methods**

**Determination of fixation points in Experiment 2**

The positions of two of the designated body regions to be fixated (torso and pelvis) were determined from a prior pilot study of Experiment 1. The peak vertical gaze density was first determined for upright and inverted whole and headless bodies separately (see Figure A). The vertical coordinate of the torso fixation point was calculated as the average of the peak vertical coordinates between upright whole and headless body trials. The vertical coordinate of the pelvis fixation point was calculated as the average of the peak vertical coordinates between inverted whole and headless body trials. Given the evidence in favor of the importance of head posture information in the body posture discrimination task (Brandman & Yovel, 2010, 2012; Yovel, Pelc, & Lubetzky, 2010), the head region was also added to the possible designated body regions. The vertical coordinate of the head fixation point was placed at the face area of the body resulting from the average of all the whole body stimuli. The horizontal coordinate for all three designated body regions was the midline of the body resulting from the average of all the whole body stimuli.

***Figure A. Vertical profile densities for the five pilot participants.*** *The curves (not normalized) visualize eye-movement density over specific vertical body features and represent the spatial densities of eye-movements summed across the vertical dimension for each condition.*

**Areas of interest (AOIs)**

Identical rectangular areas-of-interest (AOIs) were applied around the head region of all body stimuli, including the headless bodies, (Figure B, for an example) using EyeLink Data Viewer software. These AOIs were never visible to participants during the experiment.


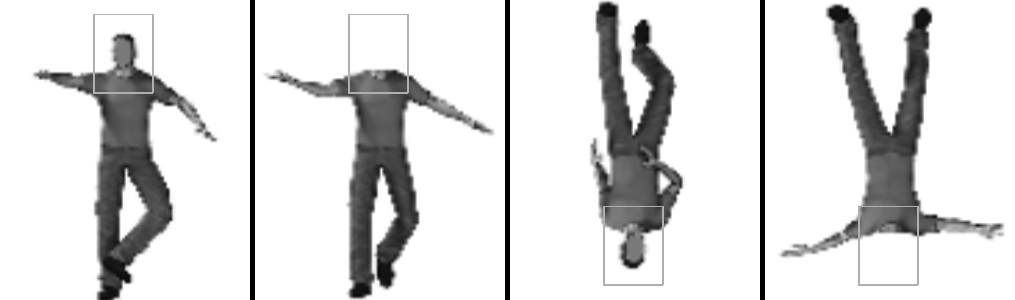


***Figure B. Head AOI on an example body stimulus.*** *The head AOI was identical in size, shape, and location (relative to the body) across all trials.*

**Preferred location analyses**

Profile density plots were produced for individual participants and the absolute maxima in the pre-normalized vertical profile density plots were utilized to designate each participant’s preferred gaze locations for upright and inverted whole body conditions. Maxima above (within an upright body reference frame) the neck were designated as “head”, above the pant-line but below the neck as “torso”, below the pant-line but above the mid-thigh as “pelvis”, and below the knee as “feet”. Note that in many participants, there was more than one local maximum, which may have had comparable density values, and so a participant’s preferred gaze location reflects only the absolute maximum.

**Results**

**Free Viewing**

**Response Bias (Criterion C)**

Response bias (Figure C) was more conservative (i.e., lower tendency to respond “different posture” when in doubt) for upright headless trials than for the other conditions in Experiment 1. A two-way ANOVA on criterion c from Experiment 1 with Headedness (whole body, headless), Orientation (upright, inverted) as within-subject factors revealed a main effect of Headedness (F(1,15) = 5.70, p < 0.032, η_p_^2^ = 0.28) driven by a lower (i.e., more liberal) criterion for responding “different posture” for whole than headless bodies. A marginal main effect of Orientation (F(1,15) = 4.48, p = 0.052, η_p_^2^ = 0.23) was also observed, and was driven by a higher (i.e., more conservative) criterion for responding “different posture” for upright than inverted bodies. A significant interaction between Headedness and Orientation was observed (F(1,15) = 7.57, p < 0.016, η_p_^2^ = 0.34). This interaction (as well as the main effects) was driven by the criterion for upright headless bodies being higher (i.e., more conservative) than all other conditions (all three comparisons: paired t(15) > 2.77, p < 0.014 two-tailed). The other three conditions were not significantly different from one another (all three comparisons: paired t(15) < 0.45, p > 0.66 two-tailed).

***Figure C. Response bias (Criterion c) in Experiment 1 (free viewing).*** *Criterion for upright headless bodies more conservative for responding “different posture” than all other conditions and was more conservative than the ideal observer (* = p < 0.05, ** = p < 0.01, *** = p < 0.001). Error bars indicate standard error of the mean.*

For upright headless trials only, participants applied criteria that were more conservative than optimal. Criterion scores were significantly higher (one-sample t(15) = 2.82, p < 0.014 two-tailed) than the ideal observer (i.e., criterion c = 0). For all other conditions, our participants’ criteria did not significantly differ from the ideal observer (all three: one-sample t(15) < 0.78, p > 0.45 two-tailed).

**Reaction Time**

Reaction time (Figure D) was lower for upright than inverted body trials in Experiment 1. A two-way ANOVA on reaction times from Experiment 1 (i.e., the free viewing experiment) with Headedness (whole body, headless) and Orientation (upright, inverted) as within-subject factors revealed only a main effect of Orientation (F(1,15) = 29.04, p < 0.0005, η_p_^2^ = 0.66) driven by lower reaction time for upright body trials. Neither a main effect nor an interaction involving Headedness was observed (both: F(1,15) < 2.06, p > 0.17, η_p_^2^ < 0.13).

***Figure D. Reaction time in Experiment 1 (free viewing).*** *Reaction time for upright body trials was lower than that for inverted body trials (* = p < 0.05). Error bars indicate standard error of the mean.*

**Head Area of Interest (AOI) Analyses**

We measured the proportions of trials during which at least one fixation landed within the head AOI. Headedness and Orientation independently influenced the proportion of trials with head region fixations such that the proportion was higher for whole than headless body trials and higher for upright than inverted body trials (Figure E). However, specifically fixating the head region did not seem necessary for extraction of the head posture information of whole bodies, and so differences in fixation specifically at the head region did not strongly drive the BIE.

***Figure E. Proportion of trials with at least one fixation within a head area of interest.*** *The overall proportion of trials with at least one head AOI landing was low and the magnitudes of the inversion effect in these proportions did not correlate with those in discrimination (d’) performance across participants (whole body trials: ρ_Spearman’s_ (15) = 0.19, p > 0.47; headless bodies: ρ_Spearman’s_(15) = -0.23, p > 0.41), suggesting that differences in fixating specifically at the head region are not strongly driving the inversion effect (* = p < 0.05, ** = p < 0.01, *** = p < 0.001). Error bars indicate standard error of the mean.*

A two-way ANOVA on proportion of trials with at least one head AOI landing with Headedness (whole body, headless) and Orientation (upright, inverted) as within-subject factors revealed a main effect of Headedness (F(1,15) = 45.39, p < 0.0005, η_p_^2^ = 0.75) driven by higher a proportion for whole body trials and a main effect of Orientation (F(1,15) = 14.36, p < 0.0025, η_p_^2^ = 0.49) driven by a higher proportion for upright body trials. No interaction between Headedness and Orientation was present (F(1,15) = 0.20, p > 0.66, η_p_^2^ = 0.013).

Interestingly, however, the overall proportion of trials with at least one head AOI landing was low. The condition with the highest proportion (upright whole bodies) had on average only about 55% of trials receiving at least one fixation to the head region. Additionally, while there was an inversion effect seen in these proportions that might suggest a relationship of head region fixation with discrimination performance that could have driven the body inversion effect, the magnitudes of the inversion effect in proportions did not correlate with those in discrimination (d’) performance across participants (whole body trials: ρ_Spearman’s_ (15) = 0.19, p > 0.47; headless bodies: ρ_Spearman’s_(15) = -0.23, p > 0.41). This suggests that differences in fixating at the head region are not strongly driving the inversion effect. Specifically fixating the head region may not be necessary for adequate utilization of the head posture information during the task in the case of whole bodies.

**Distribution of Gaze in the x-dimension**

Participants’ gaze predominantly landed near the midline of the body, though with slightly more density on left-sided body extremities (screen reference frame) for headless than for whole body conditions, and slightly more to the right of midline for whole compared to headless bodies.

The profile density (see Methods and Supplementary Methods) of gaze along the x-axis (Figure F) indicated a group-level peak near the midlines of the bodies with rapidly declining density away from midline for all conditions. This suggests that participants’ gaze fell near the midline of the body more so than at the extremities. No significant differences were observed between upright and inverted conditions for either whole or headless bodies (Figure G). However, the significantly weaker decline in density out to the extremities on the left side for headless body conditions reveals that left-sided extremities were gazed at more often for headless bodies than for whole body conditions. Also the significantly weaker decline in density to the right of midline on the right side for whole body conditions reveals that the right side of midline was gazed toward more often for whole than for headless body conditions (Figure H).

***Figure F.*** ***Horizontal profile densities.*** *The curves visualize relative eye-movement density over specific horizontal positions along the body and represent the spatial densities of eye-movements summed along the vertical dimension for each condition.*

***Figure G.*** ***Horizontal profile density statistical contrasts between upright and inverted body trials.*** *The curves in separate plots for* ***(A)*** *whole and* ***(B)*** *headless body trials represent the horizontal profile densities for the upright and inverted body trials. Horizontal positions at which upright body density was statistically significantly greater (q < 0.05) than inverted body density are indicated in red shading of the curves and images. The inverse is indicated in blue.*

***Figure H.*** ***Horizontal profile density statistical contrasts between whole and headless body trials.*** *The curves in separate plots for* ***(A)*** *upright and* ***(B)*** *inverted body trials represent the horizontal profile densities for the whole and headless body trials. Horizontal positions at which whole body density was statistically significantly greater (q < 0.05) than headless body density are indicated in red shading of the curves and images. The inverse is indicated in blue.*

**Distribution of Gaze with Larger Sample Size (n = 27)**

We repeated analyses of gaze distribution with larger participant sample sizes. Importantly, the gaze density patterns and statistical contrast results are similar to those of the smaller sample size included in the main manuscript. The additional data for these analyses come from those participants who were excluded from all other analyses due to scheduling issues or failure to maintain fixation in Experiment 2, but whose eye-tracking data quality in Experiment 1 was not compromised. In each of the whole and headless conditions, there are a total of 27 participants. Four participants in each whole/headless condition only participated in the given condition and so are not shared between the whole/headless conditions. Therefore, only upright vs. inverted statistical contrasts were conducted, given that our analyses are within-subject contrasts.

***Figure I. Vertical profile densities with 27 participants.*** *The curves visualize relative eye-movement densities over specific vertical positions along the body and represent the spatial densities of eye-movements summed along the horizontal dimension for each condition.*

***Figure J. Vertical profile density statistical contrasts between upright and inverted body trials with 27 participants.*** *The curves in separate plots for* ***(A)*** *whole and* ***(B)*** *headless body trials represent the vertical profile densities for the upright and inverted body trials. For reference, the head is displayed in the headless bodies plot, even though it was absent in the actual stimuli. Vertical positions at which upright body density was statistically significantly greater (q < 0.05) than inverted body density are indicated with red shading of the curve and image. The inverse is indicated in blue.*

***Figure K.*** ***Horizontal profile densities with 27 participants.*** *The curves visualize relative eye-movement density over specific horizontal positions along the body and represent the spatial densities of eye-movements summed along the vertical dimension for each condition.*

***Figure L.*** ***Horizontal profile density statistical contrasts between whole and headless body trials with 27 participants.*** *The curves in separate plots for* ***(A)*** *upright and* ***(B)*** *inverted body trials represent the horizontal profile densities for the whole and headless body trials. For reference, the head is displayed in the headless bodies plot, even though it was absent in the actual stimuli. Horizontal positions at which whole body density was statistically significantly greater (q < 0.05) than headless body density are indicated in red shading of the curves and images. The inverse is indicated in blue.*

**Distributions of Gaze by Gender of Observers**

We analyzed the gaze distributions by gender of observers separately for each experimental condition in order to investigate whether there were any gaze pattern differences between genders of observers. This was conducted as an exploratory analysis only, and a future study specifically designed for testing any potential gender of observer differences would be warranted to reach more definitive conclusions. This analysis yielded no notable gender differences in our participant sample. There was only one short vertical segment of rather low magnitude difference for upright whole bodies. Note that for this analysis, we again included the aforementioned pool of additional participants so that the number of males in the analysis would be larger. From this pool, there are 6 additional males who participated in at least one of the two (whole/headless) experimental sessions. Of these, three males participated in both sessions, two in only the headless session, and one in only the whole body session. Therefore, there are a total of 9 male participants included in the headless body plot, and 8 in the whole body plot.

Additionally, these gender-based profile density contrast analyses were not conducted identically in procedure to the other profile density contrast analyses reported in this study. This is because gender contrasts could not be conducted within-subject as the other contrasts could and because there were not equal numbers of participants between the genders. Therefore, contrasts were between the genders’ average profile densities. The permutation tests assumed exchangeability between genders of observers under the null hypothesis of equivalence of gaze profile density shapes between observers, and the proportion of resampled observers assigned to each “gender” label in each iteration was identical to the proportion of male and female observers in the actual data. Besides these differences, the area-normalization of gaze profile density, the number of resampling iterations, the procedure for calculating statistical significance with the resampled data, and the corrections for multiple corrections were the same as all the other profile density contrast analyses reported in this study.

***Figure M. Vertical profile density statistical contrasts between male and female participants.*** *The curves in separate plots for* ***(A)*** *upright whole* ***(B)*** *inverted whole* ***(C)*** *upright headless and* ***(D)*** *inverted headless body trials represent the vertical profile densities for the male and female participants. For reference, the head is displayed in the headless bodies plot, even though it was absent in the actual stimuli. Vertical positions at which upright body density was statistically significantly greater (q < 0.05) than inverted body density are indicated with red shading of the curve and image. The inverse is indicated in blue.*

***Figure N.*** ***Horizontal profile density statistical contrasts between whole and headless body trials with 27 participants.*** *The curves in separate plots for* ***(A)*** *upright whole* ***(B)*** *inverted whole* ***(C)*** *upright headless and* ***(D)*** *inverted headless body trials represent the horizontal profile densities for the male and female participants. For reference, the head is displayed in the headless bodies plot, even though it was absent in the actual stimuli. Horizontal positions at which whole body density was statistically significantly greater (q < 0.05) than headless body density are indicated in red shading of the curves and images. The inverse is indicated in blue.*

**Distribution of Gaze in the y-dimension for the First Stimulus**

The fixation patterns reported in the main manuscript are for the second stimulus only. This is because the duration of the first stimulus was brief (250 msecs), so participants would not have had time to make many, if any, saccades during first stimulus presentations. Therefore, first stimulus gaze patterns would not necessarily reflect the participants’ response to stimulus orientation, which was unpredictable across trials. However, gaze patterns on the first stimulus can reveal how participants preferred to preemptively place their gaze before the stimuli appeared. Therefore, to determine this, we also analyzed gaze patterns for the first stimulus for the 16 participants included in the study (Figures O-T).

For first stimuli, gaze density was overwhelmingly located at the torso of upright bodies, and at the thigh region of inverted bodies. This pattern of gaze differences between upright and inverted bodies was statistically significant (Figure P). These preferred gaze locations indicate that participants preemptively placed gaze slightly above the screen-centric vertical center of the expected stimulus.

There were also some statistically significant differences in vertical profile density between whole and headless bodies during the first stimulus (Figure Q). These difference patterns were driven simply by a slightly less skew toward the screen-centric lower half of the stimulus for headless compared to whole bodies. This was likely due to the vertical extent of the stimuli being shorter for headless than whole bodies and participants being able to expect this, given that whole and headless trials were in separate experimental sessions. There was also a low-magnitude difference at the head region between whole and headless bodies. Unsurprisingly, this difference was a slightly greater gaze density at the head region for whole than headless bodies.

***Figure O. Vertical profile densities for the first stimulus.*** *The curves visualize relative eye-movement densities over specific vertical positions along the body and represent the spatial densities of eye-movements summed along the horizontal dimension for each condition.*

***Figure P. Vertical profile density statistical contrasts between upright and inverted body trials for the first stimulus.*** *The curves in separate plots for* ***(A)*** *whole and* ***(B)*** *headless body trials represent the vertical profile densities for the upright and inverted body trials. For reference, the head is displayed in the headless bodies plot, even though it was absent in the actual stimuli. Vertical positions at which upright body density was statistically significantly greater (q < 0.05) than inverted body density are indicated with red shading of the curve and image. The inverse is indicated in blue.*

***Figure Q. Vertical profile density statistical contrasts between whole and headless body trials for the first stimulus.*** *The curves in separate plots for* ***(A)*** *upright and* ***(B)*** *inverted body trials represent the vertical profile densities for the whole and headless body trials. Vertical positions at which whole body density was statistically significantly greater (q < 0.05) than headless body density are indicated with red shading of the curve and image. The inverse is indicated in blue.*

***Figure R.*** ***Horizontal profile densities for the first stimulus.*** *The curves visualize relative eye-movement density over specific horizontal positions along the body and represent the spatial densities of eye-movements summed along the vertical dimension for each condition.*

***Figure S.*** ***Horizontal profile density statistical contrasts between upright and inverted body trials for the first stimulus.*** *The curves in separate plots for* ***(A)*** *whole and* ***(B)*** *headless body trials represent the horizontal profile densities for the upright and inverted body trials. Horizontal positions at which upright body density was statistically significantly greater (q < 0.05) than inverted body density are indicated in red shading of the curves and images. The inverse is indicated in blue.*

***Figure T.*** ***Horizontal profile density statistical contrasts between whole and headless body trials for the first stimulus.*** *The curves in separate plots for* ***(A)*** *upright and* ***(B)*** *inverted body trials represent the horizontal profile densities for the whole and headless body trials. Horizontal positions at which whole body density was statistically significantly greater (q < 0.05) than headless body density are indicated in red shading of the curves and images. The inverse is indicated in blue.*

**Forced Fixation**

**d’**

***Figure U.*** ***Discrimination performance (d’) for all conditions in Experiment 2 (forced fixation).*** *Two-way interactions of Headedness and Orientation and of Headedness and Location were present (see Figure 6); however, no three-way interaction among Headedness, Orientation, and Location was observed.*

***Figure V. BIE magnitudes in Experiment 2 (forced fixation).*** *The magnitude of the BIE was smaller for headless than whole bodies; however, fixation at different body locations did not modulate the magnitude of the body inversion effect*

**Response Bias (Criterion C)**

Response bias differed independently by Headedness and by Location (Figure W). A three-way ANOVA on criterion c from Experiment 2 with Headedness (whole body, headless), Orientation (upright, inverted), and Location (head, torso, pelvis) as within-subject factors revealed a main effect of Headedness (F(1,15) = 7.63, p < 0.016, η_p_^2^ = 0.34) driven by a lower (i.e., more liberal) criterion for responding “different posture” for whole than headless bodies. A main effect of Location (F(2,30) = 5.36, p < 0.011, η_p_^2^ = 0.26) was also observed, and was driven by a lower (i.e., more liberal) criterion for responding “different posture” for head-focused trials than for torso-focused trials (paired t(15) < 0.02, two-tailed, bias corrected G_Hedges_ = 0.29; the other two comparisons: paired t(15) > 0.10, two-tailed). No main effect of Orientation was detected nor were any interactions (all p > 0.17).

***Figure W.*** ***Response bias (Criterion c) in Experiment 2 (forced fixation).*** *A more liberal criterion for responding “different posture” for whole than headless bodies was observed. An independent effect of a more liberal criterion for head-focused trials than for torso-focused was also observed. Also, for upright headless trials and for pelvis-focused inverted headless trials, participants applied criteria that were more conservative than the ideal observer (* = p < 0.05). Error bars indicate standard error of the mean.*

For upright headless trials and for pelvis-focused inverted headless trials, participants applied criteria that were more conservative than optimal. For upright headless trials (all three body locations: one-sample t(15) > 2.94, p < 0.011 two-tailed) and for pelvis-focused inverted headless trials (one-sample t(15) = 2.31, p < 0.037 two-tailed), our participants performed the discrimination task with a significantly higher (i.e., more conservative) criterion than the ideal observer (i.e., criterion c = 0). For all other conditions, our participants’ criteria did not significantly differ from the ideal observer (all: one-sample t(15) < 1.74, p > 0.10 two-tailed). The conservative criteria for upright headless trials in Experiment 2 conceptually replicate the conservative criterion for upright headless trials observed in Experiment 1.

**Reaction Time**

In Experiment 2, Orientation and Headedness independently influenced reaction times such that reaction time was lower for upright than inverted body trials, and lower for whole than headless body trials (Figure X). A three-way ANOVA on reaction times from Experiment 2 with Headedness (whole body, headless), Orientation (upright, inverted), and Location (head, torso, pelvis) as within-subject factors revealed main effects of Headedness (F(1,15) = 7.43, p < 0.017, η_p_^2^ = 0.33) and Orientation (F(1,15) = 28.38, p < 0.0005, η_p_^2^ = 0.65) driven by shorter reaction time for whole than headless bodies and for upright than inverted bodies. No main effect of Location was detected (F(1.32,19.79) = 2.78, p > 0.10 Greenhouse-Geisser corrected, η_p_^2^ = 0.16), nor were any interactions (all p > 0.14). The lack of significant interactions involving both Orientation and Location suggest that fixating at different body locations did not modulate the body inversion effect seen in reaction time (Figure X).

***Figure X.*** ***Reaction time in Experiment 2 (forced fixation).*** *Orientation and Headedness independently influenced reaction times such that reaction time was lower for upright than inverted body trials, and lower for whole than headless body trials (* = p < 0.05). Error bars indicate standard error of the mean.*

***Figure Y. BIE magnitudes in reaction time in Experiment 2 (forced fixation).*** *Differences in reaction time between upright and inverted bodies for each body location. Fixating at different body locations did not significantly modulate the magnitude of the body inversion effect seen in reaction time. Error bars indicate standard error of the mean.*

***Figure Z. Numbers of pixel changes between image pairs.*** *The average number of body to (white) background or background to body pixel changes between stimulus image pairs* ***(A)*** *at each vertical image coordinate, and* ***(B)*** *above the vertical midline of the stimulus images versus below the vertical midline of the stimulus images. The vertical midline of the stimulus images was approximately at the boundary of the shirt and trousers of the body stimuli. Error bars represent standard deviations.*

We quantified the number of pixels at each vertical image coordinate that changed either from body to (white) background or from background to body due to a body stimulus posture change (Figure Za). We then statistically compared the total numbers of pixels that changed between the upper (above waist) and lower (below waist) half of the body images (Figure Zb show the corresponding bar plots). The results indicate that across the image pairs there were significantly more pixel changes in the lower body compared to the upper body (paired t(17) = -6.09, p = 0.000012, two-tailed). On average, there were 5960 more pixels changing in the lower body than in the upper body. Nonetheless, our participants preferred to gaze at the upper body, indicating that these low-level image differences did not drive their gaze patterns and do not account for the apparently greater salience of the upper body in the posture discrimination task.

***Figure AA. Individual*** ***vertical profile densities.*** *The curves (not normalized) visualize eye-movement density over specific vertical positions along the body and represent the spatial densities of eye-movements summed along the horizontal dimension for each condition. All plots are in the same scale and not normalized so that absolute differences in profile density among participants are apparent.*

**References**

Arizpe, J., Kravitz, D. J., Walsh, V., Yovel, G., & Baker, C. I. (2016). Differences in Looking at Own- and Other-Race Faces Are Subtle and Analysis-Dependent: An Account of Discrepant Reports. *PloS One*, *11*(2), e0148253. doi:10.1371/journal.pone.0148253

Brandman, T., & Yovel, G. (2010). The body inversion effect is mediated by face-selective, not body-selective, mechanisms. *The Journal of Neuroscience : The Official Journal of the Society for Neuroscience*, *30*(31), 10534–10540. doi:10.1523/JNEUROSCI.0911-10.2010

Brandman, T., & Yovel, G. (2012). A face inversion effect without a face. *Cognition*, *125*(3), 365–372. doi:10.1016/j.cognition.2012.08.001

Chan, A., Silson, E., & Baker, C. (2015). Understanding the topography of face and body selectivity in human ventral temporal cortex. *Journal of Vision*, *15*(12), 623. Retrieved from http://dx.doi.org/10.1167/15.12.623

Chan, A. W.-Y., Kravitz, D. J., Truong, S., Arizpe, J., & Baker, C. I. (2010). Cortical representations of bodies and faces are strongest in commonly experienced configurations. *Nature Neuroscience*, *13*(4), 417–418.

Cox, R. W. (1996). AFNI: software for analysis and visualization of functional magnetic resonance neuroimages. *Computers and Biomedical Research, an International Journal*, *29*(3), 162–173. doi:10.1006/cbmr.1996.0014

Yovel, G., Pelc, T., & Lubetzky, I. (2010). It’s all in your head: why is the body inversion effect abolished for headless bodies? *Journal of Experimental Psychology. Human Perception and Performance*, *36*(3), 759–767. doi:10.1167/9.8.460
